# Transposable elements are enriched within or in close proximity to xenobiotic-metabolizing cytochrome P450 genes

**DOI:** 10.1186/1471-2148-7-46

**Published:** 2007-03-23

**Authors:** Song Chen, Xianchun Li

**Affiliations:** 1 Department of Entomology and BIO5, University of Arizona, Tucson, AZ 85721, USA

## Abstract

**Background:**

Transposons, i.e. transposable elements (TEs), are the major internal spontaneous mutation agents for the variability of eukaryotic genomes. To address the general issue of whether transposons mediate genomic changes in environment-adaptation genes, we scanned two alleles per each of the six xenobiotic-metabolizing *Helicoverpa zea *cytochrome P450 loci, including *CYP6B8*, *CYP6B27*, *CYP321A1*, *CYP321A2*, *CYP9A12v3 *and *CYP9A14*, for the presence of transposon insertions by genome walking and sequence analysis. We also scanned thirteen *Drosophila melanogaster *P450s genes for TE insertions by *in silico *mapping and literature search.

**Results:**

Twelve novel transposons, including LINEs (long interspersed nuclear elements), SINEs (short interspersed nuclear elements), MITEs (miniature inverted-repeat transposable elements), one full-length transib-like transposon, and one full-length Tcl-like DNA transpson, are identified from the alleles of the six *H. zea *P450 genes. The twelve transposons are inserted into the 5'flanking region, 3'flanking region, exon, or intron of the six environment-adaptation P450 genes. In *D. melanogaster*, seven out of the eight *Drosophila *P450s (*CYP4E2*,* CYP6A2*,* CYP6A8*,* CYP6A9*, *CYP6G1*,* CYP6W1*,* CYP12A4*,* CYP12D1*) implicated in insecticide resistance are associated with a variety of transposons. By contrast, all the five *Drosophila *P450s (*CYP302A1*,* CYP306A1*,* CYP307A1*, *CYP314A1 *and *CYP315A1*) involved in ecdysone biosynthesis and developmental regulation are free of TE insertions.

**Conclusion:**

These results indicate that TEs are selectively retained within or in close proximity to xenobiotic-metabolizing P450 genes.

## Background

All organisms must adapt to their environments composed of biotic and abiotic factors to survive and reproduce. This requires organisms to allocate a substantial portion of their genomes to encode environment response genes that can be defined as those involved in interactions external to the organisms [1]. Examples of environmental response genes are those involved in pathogenesis or virulence (in pathogens), biosynthesis of toxic compounds (in plants), and detoxification (in animals). To cope with the ever-changing environment, organisms are also required to have greater genomic plasticity in the environmental response genes so that novel adaptive genomic changes (mutations) are available for natural selection.

Although there may be a few environmental stress factors such as UV radiation and mutagenic xenobiotics that can directly cause genomic changes, the majority of environmental factors act as pure selection agents rather than mutagens. Therefore, the genomic plasticity necessary for coping with the ever-changing environment in these loci should arise primarily from internal spontaneous alteration events [2].

There are two possible internal mutators that can cause mutational genomic changes: error-prone DNA polymerase and transposable element (TE). In normal conditions, the error rate of DNA polymerase and the transpositional activity of TE are relatively low. Under environmental stress, both the error rate of DNA polymerase and the transpositional activity of TE transposition can be increased [3-8]. For example, the transpositional activity of TEs in plants is relatively silent during normal development but is activated by stresses, such as wounding, pathogen attack, and cell culture [4].

Enhanced TE transpositional activity may insert TEs into any loci as long as they have the corresponding target site sequence for a given TE. The retention and accumulation of TE insertions, however, will likely be loci specific. In theory, nature would likely retain TEs inserted into the environmental response loci and/or their flanking regions but remove TEs inserted into the critical housekeeping gene loci and/or their nearby regions. Environmental response genes need a greater mutation rate and genomic plasticity to cope with the ever-changing environment, whereas essential housekeeping genes require a high fidelity because of functional constrains. Moreover, deleterious mutations within or around the essential genes, which are much more common than advantageous mutations, often result in catastrophic lethality. By contrast, deleterious mutations within or around the environmental response genes usually lead to reduction in fitness. Such differential consequences resulting from deleterious mutations are determined by the nature of the two groups of genes as well as the presence of duplicated redundant copies of the environmental response genes in the genome [1]. By retention and accumulation of TEs, the environmental response genes can gain TE-introduced selectively advantageous variations otherwise not easily available to respond successfully to changes in environment.

Several recent reports appear to support this hypothesis. Wagner et al. (2003) found that TEs are often excluded from the HOX genes, a family of linked transcription factors sharing a DNA-binding domain and playing important roles in animal body plan development in vertebrates [9]. Simons et al. (2006) identified 860 conserved TE-free regions (TFR) over 10 kb in length in the human and mouse genomes [10]. These TFR are significantly associated with genes encoding transcription factors and developmental regulators [10], consistent with the notion that TE insertions into essential housekeeping loci are strongly selected against. By the same token, Grover et al. (2003) showed that *Alu *elements are more clustered in genes involved in metabolism, transport, and signalling processes (facilitating the adaptation of signal transduction pathways to environmental changes) than in genes encoding information pathway components and structural proteins [11]. Analyses of human and mouse genomes indicated that TEs are significantly enriched in rapidly-evolving gene classes, such as those involved in immunity and response to external stimuli but are excluded from mRNAs of highly conserved genes with basic housekeeping functions in development, transcription, replication, cell structure and metabolism [12,13]. Along the same line, at least seventeen TEs are associated with the rice Xa21 gene cluster, which confers disease resistance against *Xanthomonas oryzae *[14]. In the common morning glory (*Ipomoea purpurea*), a remarkable variety of mobile elements reside in the chalcone synthase D locus (CHS-D), which encodes a key enzyme controlling the first committed step in the flavonoid biosynthetic pathway producing a wide range of compounds important in UV protection and defense against plant disease and herbivores [15]. Other than the above-mentioned differential retention of TEs between essential housekeeping genes and environmental response genes, *Tos17 *retrotransposon prefers to insert into disease/defense-related and signal transduction (kinase) genes in the rice genome [16].

Cytochrome P450 monooxygenases (P450s) are ubiquitous in virtually all living organisms and constitute a multigene family replete with gene duplication and conversion events [17]. Multiple P450 genes exist in a given genome, some of which may function as essential housekeeping genes involved in the biosynthesis of hormones [18,19]; others may act as environmental response genes involved in the biosynthesis of toxic compounds for plant defense or pathogens attack or in the detoxification of naturally-occurring and synthetic xenobiotics [2,17,20]. To address the issue of whether TEs mediate genomic changes in the environmental response P450 genes, we scanned two alleles (one from a laboratory colony, another from a cell line) per each of the six xenobiotic-metabolizing *Helicoverpa zea *P450 genes, *CYP6B8*,* CYP6B27, CYP9A12v3*,* CYP9A14*, *CYP321A1 *and *CYP321A2 *[21-25], for the presence of TE insertions by genome walking and sequence analysis. Twelve novel TEs, including LINEs (long interspersed nuclear elements), SINEs (short interspersed nuclear elements), MITEs (miniature inverted-repeat transposable elements), one full-length transib-like transposon, and one full-length Tcl-like DNA transpson, are identified from their introns, exons or flanking regions. We further surveyed TE insertions among the xenobiotic-metabolizing and ecdysone- synthesizing P450 genes in *Drosophila melanogaster *genome by *in silico *mapping at the flybase [26] and literature search. Seven out of the eight insecticide resistance responsible P450s, including *CYP4E2 *[27],* CYP6A2 *[28-30],* CYP6A8 *[28],* CYP6A9 *[29],* CYP6G1 *[31-33],* CYP6W1 *[30],* CYP12A4 *[34],* CYP12D1 *[35,36], are associated with a variety of TEs. In contrast, TEs are excluded from all of the five essential *Drosophila *P450 genes (*CYP302A1*,* CYP306A1*,* CYP307A1*,* CYP314A1 *and *CYP315A1*) that are involved in ecdysone biosynthesis and developmental regulation [18,37-40]. These results indicate that TEs are selectively enriched within or in close proximity to xenobiotic-metabolizing cytochrome P450 genes.

## Results

### Transposons within the *CYP6B *gene cluster

Seven P450 genes distributed in the *CYP6B*, *CYP4M*, and *CYP321A *subfamilies have been cloned from *H. zea *[21-23]. Previous studies have demonstrated that *CYP321A1 *is highly inducible by xanthotoxin [23] and the four *CYP6B *transcripts accumulate to varying degrees in response to a range of allelochemicals naturally encountered in hostplants (xanthotoxin, indole-3-carbinol, chlorogenic acid, flavone) as well as in response to synthetic chemicals not naturally encountered (cypermethrin, phenobarbital) [21,22]. The *CYP6B *transcripts are also inducible by plant defense signalling molecules jasmonate and salicylate, allowing this species to "eavesdrop" on plant defense signals for activating detoxification systems in advance of induced biosynthesis of host plant toxins [41]. Baculovirus-mediated expression of the *CYP6B8 *and *CYP321A1 *proteins has directly demonstrated that they are capable of metabolizing a range of allelochemicals (xanthotoxin, chlorogenic acid, quercetin, flavone) and insecticides (diazinon, cypermethrin, and aldrin) [23,24].

**Figure 1:**
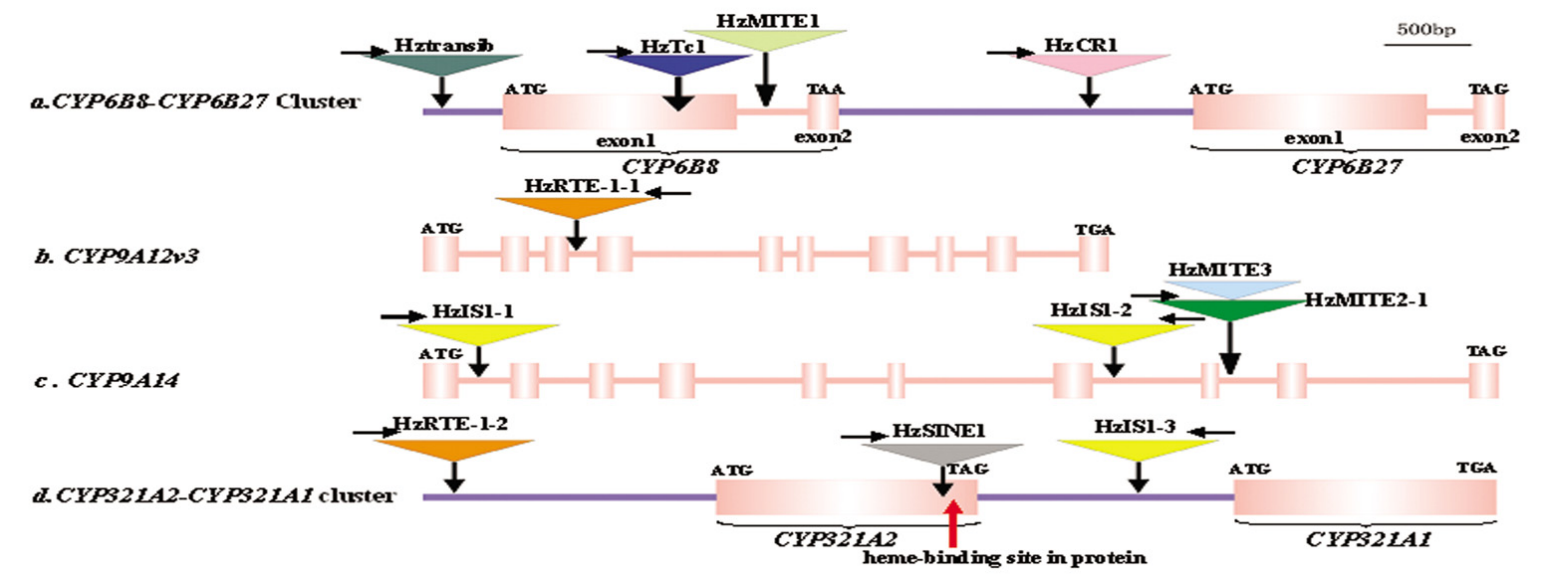
**Schematic representation of the six P450 genes and the inserted twelve transposons**. The P450 genes are shown to scale with exons depicted as filled pink boxes, introns as pink lines, and 5'/3'-flanking sequences as blue lines. The transposons are represented as colored inverted triangles with their names, orientations (horizontal arrows above triangles) and insertion positions (vertical arrows below triangles). Triangles with an identical color are different copies of one transposon. The red vertical arrow below the *CYP321A2 *indicates its heme-binding site in protein.

**Figure 2:**
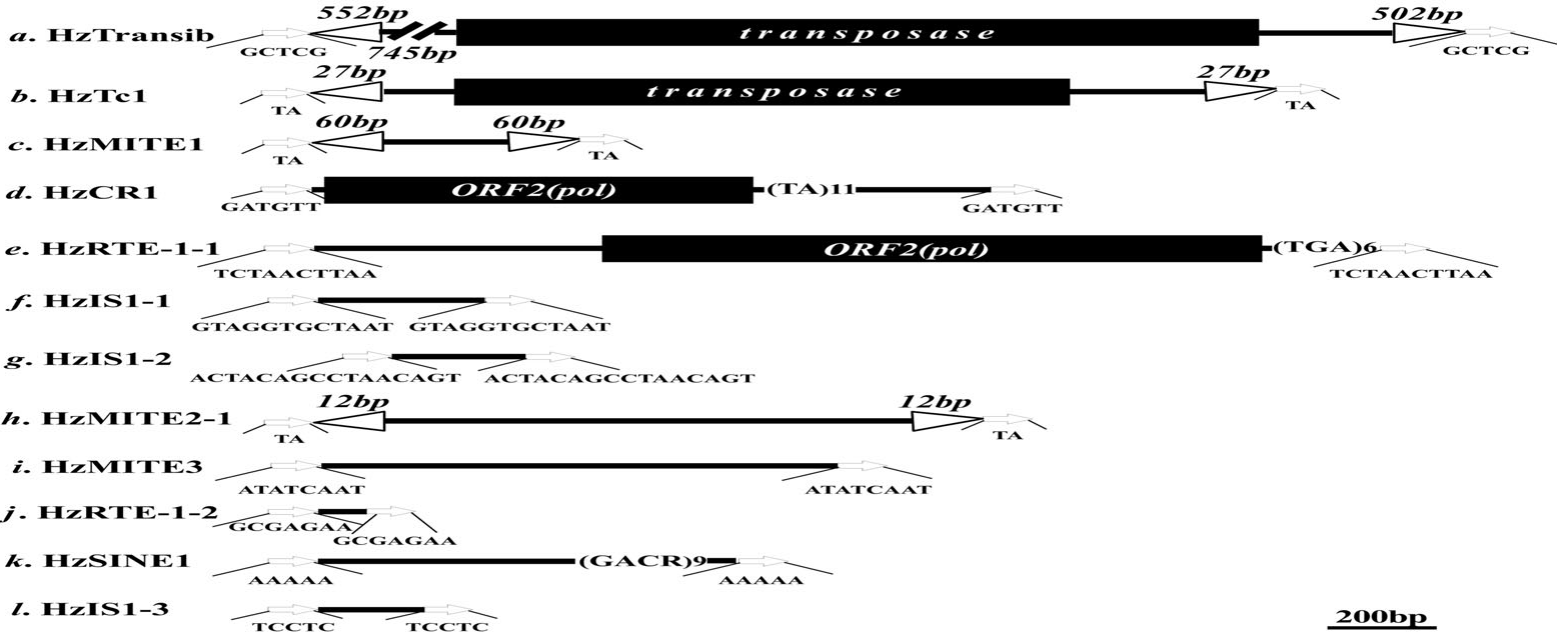
**Schematic structures of the 12 transposons**. The structure of the 12 transposons are drawn to scale (except for Hztransib1) with horizontal arrows representing putative TSDs (sequence shown underneath). Arrowheads represent TIR (length shown above), filled black boxes represent putative ORF and black lines represent non-coding sequences. Microsatellite sequences within some transposons are shown in parentheses, followed by the corresponding repeat number.

**Figure 3:**
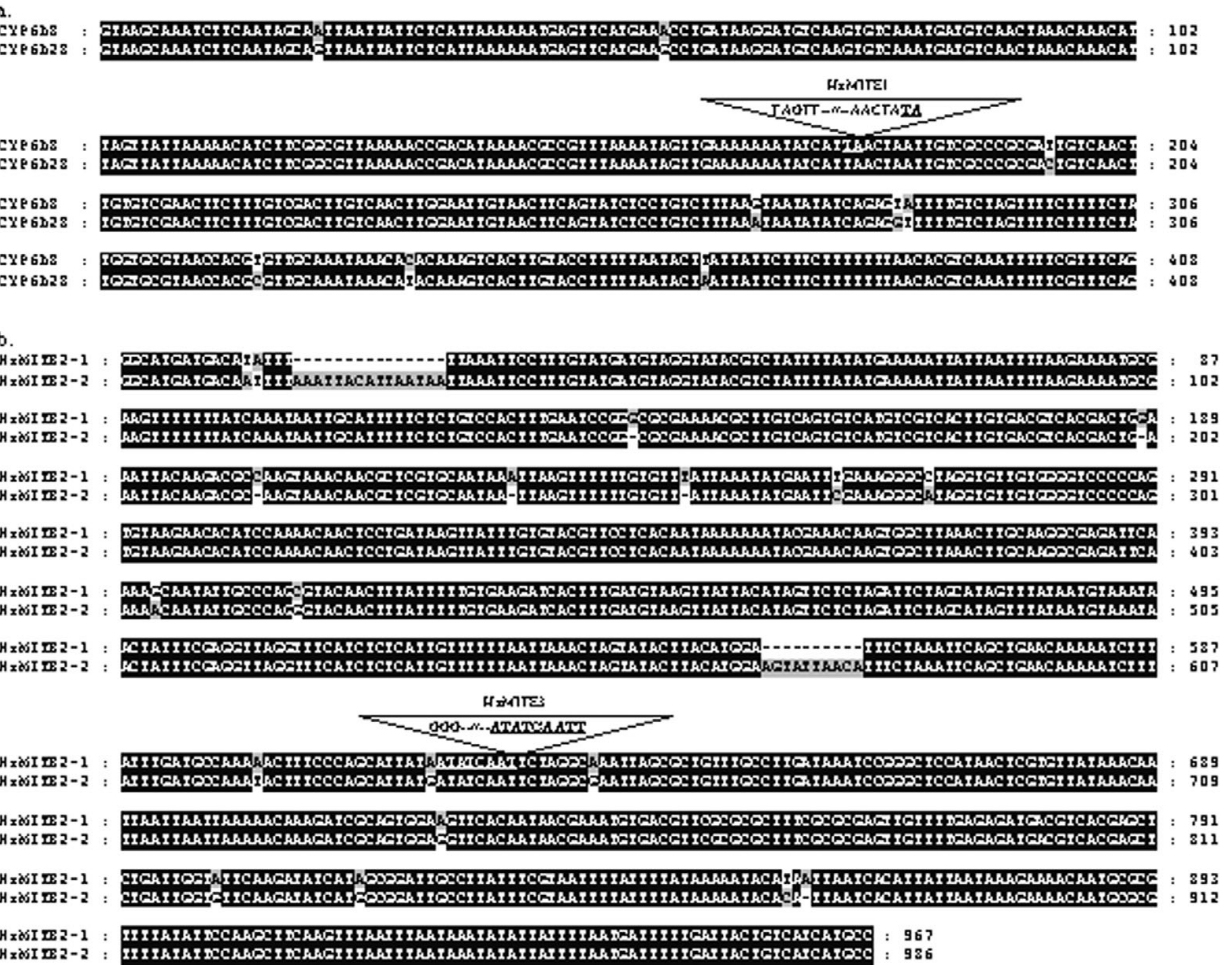
**Nucleotide alignments of *CYP6B8 *and *CYP6B28 *introns (a) and of two HzMITE2 copies (b)**. Sequence alignments were generated using the Genedoc software. HzMITE1 and HzMITE3 are indicated by inverted triangles. TSDs flanking each element are underlined and in bold. TIRs for HzMITE2-1and HzMITE2-2 are shown in italic. While HzMITE2-1 is inserted in the 8th intron of *CYP9A14*, HzMITE2-2 is inserted in the 1st intron of the *H. zea *delta-9 like acyl-CoA desaturase (HzPGDs3) in an opposite orientation. Accession numbers of *CYP6B8 *and HzPGDs3 are [GenBank: AF285186] and [GenBank: AF297109], respectively.

Here we cloned the 5'-flanking sequences of the four xenobiotic-metabolizing *H. zea CYP6B *genes by genome walking approach [see Additional file 1] and then searched for the presence of transposon insertions by sequence analysis. We successfully recovered the genomic sequences and the 5'-flanking sequences of *CYP6B8 *and *CYP6B27 *but failed to recover the 5'-flanking sequences of *CYP6B9 *and *CYP6B28 *from our current laboratory population and from the *H. zea *midgut cell line RP-HzGUT-AW1 [42]. It is possible that *CYP6B28 *is just an allele of *CYP6B8 *since it is essentially identical to *CYP6B8 *except for a 227-bp insertion in its intron (Figure 3a) [43]. Re-examination of this 227-bp insertion sequence reveals that it has a 60-bp perfect terminal inverted repeats (TIRs) flanked by 2-bp target site duplications (TSDs), lacks coding potential and is AT rich (59%). These are the common structural characteristics of MITEs [44], thus we designate it as HzMITE1 (Figure 1a; Figure 2c; Table 1).

**Table 1:** The structural characteristics of twelve transposons characterized from six xenobiotic-metabolizing P450 genes in *H. zea*

**element name**	**class**	**size, bp**	**characteristics**	**Location**	**laboratory strain**	**midgut cell line**
Hztransib	transib	3518	TIRs,5bp-TSD	*CYP6B8 *promoter region		
HzTc1	Tc1	1558	TIRs,2bp-TSD	*CYP6B8 *exon1		
HzMITE1	MITEs	227	TIRs,2bp-TSD	*CYP6B8 *intron		
HzCR1	non-LTR	1099	6-bpTSD	*CYP6B *cluster intergenic region		
HzRTE-1-1	non-LTR	1754	10-bpTSD	*CYP9A12v3 *3^rd ^intron		
HzIS1-1	-	264	13-bpTSD	*CYP9A14 *1^st ^intron		
HzIS1-2	-	393	16-bpTSD	*CYP9A14 *7^th ^intron		
HzMITE2-1	MITEs	967	TIRs,2bp-TSD	*CYP9A14 *8^th ^intron		
HzMITE3	MITEs	945	9-bpTSD	*CYP9A14 *8^th ^intron		
HzRTE-1-2	non-LTR	62	7-bpTSD	*CYP321A2 *promoter region		ND
HzSINE1	SINEs	556	5-bpTSD	*CYP321A2 *exon		
HzIS1-3	-	190	5-bpTSD	*CYP321A *cluster intergenic region		

* ND: Not detected; : present; : absent

In the laboratory population and the cell line, *CYP6B8 *and *CYP6B27 *are adjacent to each other in a head-to-tail arrangement, with *CYP6B8 *located 2030-bp upstream of *CYP6B27 *(Figure 1a). By comparing the sequence of the *CYP6B8-CYP6B27 *cluster in the laboratory colony and that in the cell line, three transposon insertions are characterized. One transposon insertion is found in the 5'-flanking promoter region of *CYP6B8 *in the midgut cell line *CYP6B8-CYP6B27 *cluster (Figure 1a; Table 1). This 3518-bp DNA transposon insertion has long TIRs and is flanked by 5-bp (GCTCG) TSDs (Figure 2a). The deduced 489 amino acids sequence has 34% identity with transib5 from *Drosophila melanogaster *[45]. Based on these features, it is designated as Hztransib. Another transposon is found in the exon1 of *CYP6B8 *in the midgut cell line (Figure 1a; Table 1). This 1558-bp transposon has typical features of Tc1 family transposon, such as 27-bp perfect TIRs flanked by 2-bp (TA) TSDs (Figure 2b). Its putative open reading frame (ORF) encodes 375 amino acids and share 23% identity with Tc1 transposon from *Caenorhabditis elegans *[46]. Thus it is designated as HzTc1.

The third one is inserted in the intergenic region of the *CYP6B8-CYP6B27 *cluster, i.e. the 5' flanking region of *CYP6B27 *or the 3'-flanking sequence of *CYP6B8 *(Figure 1a). This insertion is present in the *CYP6B8*-*CYP6B27 *cluster of the laboratory population but not in the midgut cell line (Table 1). This transposon is flanked by 6-bp TSDs (Figure 2d) and has an ORF sharing 29.1% amino acids identity with *Maui *transposon, a member of CR-1 (chicken repeat 1) family of non-LTR retrotransposons, from the pufferfish *Fugu rubripes *[47]. This retrotransposon insertion also contains microsatellite (TA dinucleotides) repeats at the 3' end but lacks a poly-A tail, which usually happened in the CR1 family of non-LTR retrotransposons [47]. Thus, we designate it as HzCR-1. Amino acids sequence alignment with other CR1 clade retrotransposons demonstrates that HzCR1 is truncated at the 5' end, losing apurinic-apyrimidic endonuclease (AP ENDO) domains and most of reverse transcriptase (RT) subdomains. Among the seven RT subdomains defined by Xiong and Eickbush [48], only the seventh subdomain and part of the sixth subdomain are retained in the HzCR1 (see Additional file 2).

### Transposons within the introns of *CYP9A12v3 *and *CYP9A14*

Two cDNA sequences representing *CYP9A12 *and *CYP9A14 *were isolated from a pyrethroid-resistant strain of *Helicoverpa armigera *[25], the old world sibling species of *H. zea*. The fact that the *CYP9A12 *and *CYP9A14 *transcripts in the pyrethroid-selected resistant strain are overexpressed 433- and 59-fold in the fatbody and 19- and 4.3-fold in the midgut, respectively, in comparison with the unselected parental strain, indicates that these two P450s are associated with pyrethroid resistance [25]. To examine if any transposon is inserted in the homologs of these two insecticide-metabolizing *H. armigera CYP9A *genes in *H. zea*, gene-specific primers (see Additional file 1) are designed to PCR-amply the genomic sequences of the *H. zea *homologs from the laboratory strain and the midgut cell line. The orthologous *CYP9A *genes obtained from the *H. zea *laboratory strain are designated as *CYP9A12v3 *(5.7 kb) and *CYP9A14 *(8.4 kb) (Nelson, personal communication). Sequence comparison with the cDNA sequences of the *H. armigera CYP9A12 *and *CYP9A14 *reveals that the two paralogous *H. zea CYP9A *genes share an identical genomic structure, each having 10 exons and 9 introns of variable sizes (Figure 1b, 1c). Their intron/exon locations and boundary sequences (all of the 9 introns follow the GT-AG rule in both *CYP9A12v3 *and *CYP9A14*) are identical.

One transposon is characterized from the *H. zea CYP9A12v3 *by using blastx against a nonredundant database. This transposon insertion, present in both the laboratory strain and the midgut cell line, is 1764-bp long, flanked by 10-bp target site duplications (TSDs), and inserted in the third intron of *CYP9A12v3 *(Figure 1b; Figure 2e; Table 1). It has one ORF that is orientated in an opposite direction with *CYP9A12v3 *(Figure 1b; Figure 2e) and encodes a reverse transcriptase with 40% amino acids sequence identity with the RTE-1 retrotransposon in *Caenorhabditis elegans *[49]. Its 3' untranslated region is unusually short and is predominantly composed of TGA trinucleotide repeats, the typical feature found in the members of the RTE clade [50] (Figure 2e). The typical feature and high amino acids sequence identity it shares with the members of the RTE-1 clade indicates that it is a RTE-1 like non-LTR retrotransposon element and thus is designated as HzRTE-1. Amino acids sequence alignment with several RTE-1 retrotransposons demonstrates that HzRTE-1 is a 5'-truncated non-LTR retrotransposon that retains most of RT domain but loses AP ENDO domains. Among the seven RT subdomains, only subdomains 3rd-7th are retained in the HzRTE-1 (see Additional file 3).

**Figure 4:**
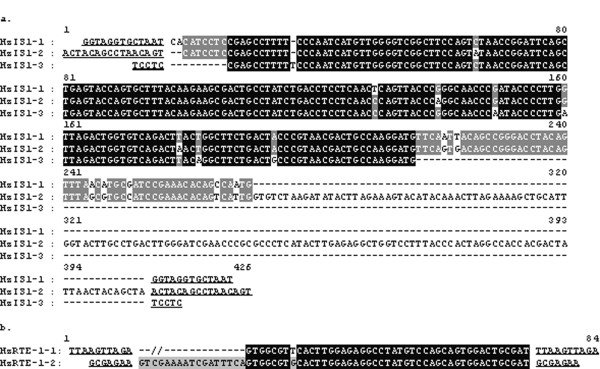
**Nucleotide alignments of three Hz IS1 copies (a) and of two HzRTE-1 copies (b)**. Sequence alignments were generated using the Genedoc software. TSDs flanking each TE are underlined and in bold.

Surprisingly, four transposons are characterized from the *CYP9A14 *locus (Figure 1c; Table 1). They are inserted into intron1, 7, and 8 of *CYP9A14*, respectively. The two elements inserted into intron 1 and 7 are 99% identical to each other, suggesting they are two copies of the same transposon. These two copies, however, have different TSDs and are oriented opposite to each other (Figure 1c; Figure 4a). Interestingly, this element is also inserted into the *CYP321A2*-*CYP321A1 *gene cluster (see below), namely, the 3'-flanking sequence of *CYP321A2 *and the 5'-flanking sequence of *CYP321A1 *(Figure 1d; Table 1). This copy also has different TSDs and is shorter in the 3' end than the two copies in the intron 1 and 7 of *CYP9A14A1 *(Figure 2f, 2g and 2l; Figure 4a). Because this element has no sequence similarity to any previously published insertion sequence and has no features that belong to Class II or Class I transposon, it is thus referred to as a TE-like element. We designate this element as HzIS1 (*H. zea *Insertion Sequence 1), with HzIS1-1, HzIS1-2 and HzIS1-3 representing its insertions in the first and seventh introns of *CYP9A14 *and the *CYP321A2*-*CYP321A1 *cluster, respectively. The laboratory strain has all three copies, whereas the midgut cell line has HzIS1-1 and HzIS1-3 only (Table 1).

In the eighth intron of *CYP9A14 *from the laboratory strain, two transposons are characterized, with one nested into another (Figure 1c; Table 1). BLAST search shows that the flanking transposon shares 96% sequence identity with the middle sequence (in an opposite orientation) of the 1^st ^intron of the *H. zea *acyl-CoA desaturase gene (HzPGDs3) (Figure 3b) [51]. In addition, these two sequences also share identical 12-bp TIRs and identical TSDs, indicating they are two copies of the same transposon (Figure 2h; Figure 3b). Because they have the common structural characteristics of MITEs [44], including small size, AT rich (67.5–67.7%), TIRs and lack of coding potential, we designate them as HzMITE2-1 (the flanking transposon in the 8^th ^intron of *CYP9A14*) and HzMITE2-2 (the copy in the intron of the HzPGDs3), respectively. The nested transposon within HzMITE2-1 (Figure 1c; Figure 3b) is 946-bp long, AT rich (67.8%), lacks coding potential, and is flanked by 9-bp TSDs (Figure 2i). These features suggest that it is also a MITE-like element although without flanking TIRs [44]. This nested transposon is designated as HzMITE3. The *CYP9A14 *allele from the midgut cell line has the flanking HzMITE2-1 only (Table 1).

### Transposons within the *CYP321A2-CYP321A1 *gene cluster

To examine if there are transposons around the *CYP321A1 *locus, another well-defined xenobiotic-metabolizing P450 in *H. zea *[23], we cloned the 5'-flanking sequence of *CYP321A1 *from both the laboratory strain and the midgut cell line. A 5.4-kb fragment upstream of *CYP321A1 *was obtained from the laboratory population by genome walking. From this fragment, we identified another P450 gene, a paralog of *CYP321A1*, is designated as *CYP321A2 *(Nelson, personal communication). Like the *CYP6B8-CYP6B27 *cluster, the *CYP321A2*-*CYP321A1 *paralogs are oriented in a head-to-tail arrangement, with *CYP321A2 *located 1467-bp upstream of *CYP321A1 *(Figure 1d). Compared with *CYP321A1 *and *CYP321A2 *alleles isolated from the midgut cells, the *CYP321A2 *allele from the laboratory population contains a 556-bp TE insertion located 126-bp upstream of its heme-binding signature motif in its ORF (Figure 1d; Table 1), resulting in a 3'-truncated transcript (Chen & Li, unpublished data). The insertion sequence is flanked by 5-bp (AAAAA) TSDs and ends with tetramer repeats (GACR) at its 3'end (Figure 2k), which is a feature found in some SINEs such as Artw and Pst elements in cows [52] and CR1 element in chickens [53]. The fact that it has no sequence similarity to any previously published transposable element suggests that it is a novel SINE element. We therefore named it as HzSINE1.

In addition, there are two other transposable elements in the *CYP321A2-CYP321A1 *cluster, one located in the intergenic region, another inserted in the 5'-flanking region of *CYP321A2 *(Figure 1d; Table 1). The transposon in the intergenic region is 99% identical to HzIS1-1 and HzIS1-2 found in the intron 1 and 7 of *CYP9A14 *(Figure 4a) and thus is designated as HzIS1-3 (see above). The transposon inserted in the 5'-flanking region of *CYP321A2 *is an extremely 5'-truncated copy of the HzRTE-1 element found in the 3^rd ^intron of *CYP9A12v3 *(see above). This HzRTE-1 copy completely loses its ORF and retains only the 62-bp 3'UTR and the flanking 7-bp TSD (Figure 2j; Figure 4b). To differentiate the two HzRTE-1 copies, we name them as HzRTE-1-1 (the one found in the 3^rd ^intron of *CYP9A12v3*) and HzRTE-1-2 (the extremely-truncated copy in the 5'-flanked promoter of *CYP321A2*).

### Differential TE insertions around *Drosophila melanogaster *P450 genes

An extensive literature search shows that thirteen out of the 90 P450 genes in the *D. melanogaster *genome have been functionally defined (Table 2) [54]. Eight of them, including *CYP4E2 *[27],* CYP6A2 *[28-30], *CYP6A8 *[28],* CYP6A9 *[29],* CYP6G1 *[31-33], *CYP6W1 *[30],* CYP12A4 *[34], *CYP12D1 *[35,36], are implicated in insecticide resistance and xenobiotic metabolism. The other five P450 genes (*CYP302A1*,* CYP306A1*,* CYP307A1*,* CYP314A1 *and *CYP315A1*) are involved in ecdysone biosynthesis and developmental regulation [18,37-40]. To test if transposons are more enriched in the xenobiotic-metabolizing *Drosophila *P450 genes as opposed to the ecdysone-synthesizing *Drosophila *P450s, we surveyed the insertions of TE within or in close proximity to the thirteen P450 loci and GSTD1, a DDT-metabolizing gene, by *in silico *mapping at the flybase [26]. Seven out of the eight insecticide resistance responsible P450 genes and the DDT-metabolizing GSTD1 are associated with a variety of TEs (Table 2). In contrast, TEs are excluded from all of the five ecdysone-synthesizing P450 genes (Table 2).

**Table 2:** Comparison of TE insertions between the xenobiotic-metabolizing and ecdysone-synthesizing P450 genes in *Drosophila melanogaster*

**Gene or gene cluster**	**Function**	TE insertion and location*
*CYP6A2*	DDT resistance [28-30]	Isfun-1-Dfun-like, 721-bp upstream
*CYP6A8*	DDT resistance [28]	Max, 255-bp upstream
*CYP6A9*	DDT resistance [29]	No
*CYP6G1*	DDT, lufenuron and neonicotinoid resistance [31-33]	Accord, 300-bp upstream of the transcription start site.
*CYP6W1*	DDT resistance [30]	Two INE-1 copies, one 1174-bp upstream, the other 1842 downstream. Further downstream are two 1360, seven INE-1, one Tc1-2, one INE-1, and one mimi-me-Dpse-like.
*CYP4E2*	DDT resistance [27]	Isfun-1-Dfun-like, 707-bp upstream, jockey2, 2274-bp downstream, and Isfun-1-Dfun-like, 2854-bp downstream.
*CYP12A4*	lufenuron resistance [34]	Bari1, 15-bp within the 3' end.
*CYP12D1*	DDT resistance [35, 36]	Two pogo copies, one 8551-bp upstream, the other 98716-bp downstream.
GSTD1	DDT dehydrochlorinase activity and DDT resistance [73].	INE-1, 2191-bp upstream
*CYP302A1*	Ecdysone biosynthesis [37, 38]	No
*CYP306A1*	Ecdysone biosynthesis [39]	No
*CYP307A1*	Ecdysone biosynthesis [40]	No
*CYP314A1*	Ecdysone biosynthesis [18]	No
*CYP315A1*	Ecdysone biosynthesis [38]	No

*: Transposons without a reference are found by *in silico *mapping at the flybase [26].

## Discussion

All living organisms, from bacteria to fungi, plants, and animals, have a remarkable degree of genetic plasticity to coevolve with their highly variable and selective environments. Plant pathogens can readily adapt to pathogen-resistant crop varieties by genomic changes at their pathogenic loci and insect pests can rapidly develop resistance to synthetic insecticides through gene amplification, overexpression, and amino acids substitutions at their detoxification loci [2]. It remains speculative how the genomic changes arise at the corresponding environmental response gene loci, simply by random mutation and sexual recombination, or by another internal spontaneous mutator?

Overwhelming evidence suggest that TEs, rather than only being "selfish junk DNA" or "DNA parasites", can act as internal spontaneous mutators to produce neutral, deleterious or advantageous effects to the fitness of the host organism [55,56]. We therefore hypothesize that TEs may be selectively enriched in environmental response genes, but relatively excluded from essential housekeeping genes, resulting in greater genomic plasticity to the former but higher conservation to the later. Indeed, data obtained from scanning the *H. zea *(Table 1) and *D. melanogaster *(Table 2) P450 genes for TE insertions as well as studies on the drug-metabolizing human P450 *CYP2D6 *[57] and the pyrethroid resistant housefly P450 *CYP6D3*-*CYP6D1 *paralogs [58,59] support this hypothesis. In *D. melanogaster*, seven out of the eight insecticide resistant P450s and the DDT-metabolizing GSTD1 are associated with a variety of TEs (Table 2). *CYP6A9*, although not directly associated with a transposon, is located in the center of the *Drosophila CYP6A *cluster, which is flanked by TEs [26]. By contrast, all the five *Drosophila *P450s involved in ecdysone biosynthesis and developmental regulation are free of TE insertions (Table 2). In the housefly *Musca domestica*, two Mariner-like elements are inserted within the *CYP6D3-CYP6D1 *cluster, with one located about 500-bp upstream of *CYP6D3 *and another located about 500-bp upstream of *CYP6D1 *[60]. In human, *CYP2D6*, one of the best-known polymorphic drug-metabolizing enzymes [57], is flanked by a 2.8 kb repeat sequence (CYP-REP element) containing an *Alu *element and a tandem 10-bp direct repeat in the wild type allele [61]. The remarkable degree of interindividual variability at the *CYP2D6 *locus is largely due to gene deletion and amplification of *CYP2D6 *generated through homologous unequal recombination of no-allelic CYP-REP elements [61,62].

In the corn earworm *H. zea*, a polyphagous herbivore capable of eating hundreds of allelochemical-containing plants and rapidly acquiring insecticide resistance, scanning two alleles per each of the six *H. zea *P450 loci (*CYP68*-*CYP6B27 *cluster, *CYP9A12v3*, *CYP9A14 *and *CYP321A2*-*CYP321A1 *cluster) (12 alleles in total) by genome walking and sequence analyses found twelve novel TEs within or in close proximity to these P450 genes (Table 1). In other words, almost every allele of these environmental response P450 genes contains a TE insertion. The twelve TEs represent virtually almost all types of transposons, including full-length DNA tansposons (Hztransib, HzTc1), MITEs (HzMITE1, HzMITE2 and HzMITE3), LINEs (HzRTE-1), and SINEs (HzSINE1), indicating these xenobiotic-metabolizing P450s can tolerate and thus retain/accumulate all types of TEs rather than one particular type of TEs. It is likely that each type of TEs may induce different genomic changes upon transposition and ectopic recombination. Consequently, these P450 loci should be genomically highly variable. This is compatible with its polyphagy and rapid acquiring of insecticide resistance, two of the most important traits contributing to the tremendous success of this key agriculture pest in a wide range of agroecosystems [63,64].

The insertion sites of the twelve TEs are also variable, two in exons, six in introns, two in the 5'-flanking sequences, and two in the intergenic region of the corresponding P450 gene clusters (Figure 1). Regardless of their potential effects on reshuffling genes and/or chromosomal domains (e.g. amplification, deletion, inversion, etc.) upon illegitimate recombination, these TE insertions *per se *will likely alter the expression and function of the corresponding P450 in different ways. HzSINE1, which inserts into the coding sequence of the *CYP321A2 *in the laboratory strain of *H. zea *(Figure 1d), will probably trigger the inactivation of *CYP321A2 *since no full-length transcripts are produced (Chen & Li, unpublished data). The four TEs that inserted into the 5'-flanking sequences or the intergenic regions of the corresponding P450 clusters may lead to overexpression of the corresponding P450s and enhancements in xenobiotic metabolism and resistance as in the case of the parallel insertions of *Accord *LTR or *Doc *non-LTR retrotransposon in the 5' regulatory region of *CYP6G1 *in *D. melanogaster *or *D. simulans *[32,33]. This possibility is further strengthened by a recent study demonstrating that insertions by various forms of a truncated on-LTR retrotransposon in the 5'-flanking sequence of *CYP51 *(encoding a 14α-demethylase) lead to overexpression of *CYP51 *and sterol demethylation inhibitor fungicide resistance in the cherry leaf spot pathogen *Blumeriella jaapii *[65]. In the case of the *D. melanogaster CYP12A4*, the insertion of *Bari1 *in the 3' UTR results in 10-fold overexpression of *CYP12A4 *but no resistance to DDT [66], indicating that DDT is not a substrate of CYP12A4. Given that overexpression of *CYP12A4 *confers resistance to lufenuron [34], it is possible the *Bari1 *insertion in the 3' UTR of *CYP12A4 *will lead to resistance to lufenuron even other insecticides or plant toxins. Nonetheless, TE insertions in the 5' or 3' flanking regulatory region of xenobiotic-metabolizing P450 loci may have little or no effects on the expression of the corresponding P450 gene and resistance to xenobiotics, depending on if they introduce or disrupt any regulatory elements. The six TEs inserted in introns could be successfully spliced out during mRNA processing, and thus have no obvious effects on the function of the corresponding P450 gene.

Alternatively, they could result in exon skipping, alternative splicing, or alternations in expression profiles if the corresponding P450 introns contain regulatory sequences as exemplified by the *Mu *insertion into an intron of the *Knotted *locus in Maize [67]. Analyses of the transcript species and expression levels of the six P450 genes as well as toxicity bioassay will be necessary to determine the exact effects of the twelve TEs insertions on the expressions and functions of the six P450s, as well as on the fitness of this species in the presence of plant defense allelochemicals and synthetic insecticides.

## Conclusion

In present study, we identified twelve novel TEs in six xenobiotic-metabolizing cytochrome P450 loci in *H.zea*. The twelve TEs represent virtually almost all types of transposons and their insertion sites are also variable. In *Drosophila*, TEs are enriched in xenobiotic-metabolizing cytochrome P450 genes, but exclude from essential cytochrome P450 genes that involved in the ecdysone biosynthesis pathway. These results present for the first time evidence that TEs are more enriched in P450 genes responsible for xenobiotic metabolism as opposed to P450 genes involved in ecdysone biosynthesis and developmental regulation.

## Methods

### Insects

A laboratory strain of *H. zea*, generously provided by Dr. May R. Berenbaum (Department of Entomology, University of Illinois at Urbana-Champaigen), was maintained in an insectary kept at 28°C with a photoperiod of 16 h light:8 h dark on a semi-synthetic control diet containing wheat germ [68]. Several 5^th ^instar larvae randomly picked from the colony were individually flash-frozen in liquid nitrogen and stored in -80°C for subsequent genomic DNA isolation.

### Cell Culture

*H. zea *midgut cell line RP-HzGUT-AW1[42], generously provided by Dr. Cynthia L. Goodman (BCIRL, USDA, ARS), was cultured in ExCell 401 serum-free medium (JR BioSciences, Lenexa, KS) supplemented with 10% heat-inactivated fetal bovine serum (FBS), 50 μg/ml streptomycin, and 50 units/ml penicillin (Sigma). The cells were harvested after 72 hour culture at 27°C by a brief centrifugation at 1,000 *g *for 2 min. The cell pellets obtained were flash-frozen in liquid nitrogen and stored in -80°C until use for DNA isolation.

### DNA Extraction and Genome Walking

Genomic DNA was isolated from the 5^th ^instar larva and cell pellets, using the procedure described in Li et al [43]. The 5'-flanking sequences of the four xenobiotic-metabolizing P450 genes, *CYP6B8*, *CYP6B27*, *CYP321A1*, and *CYP321A2*, were obtained by genome walking. In brief, genomic DNA was digested by several restriction enzymes supplied in the Universal GenomeWalker kit (Clontech, CA, USA) and then ligated to the genome walking adapters according to the manufacturer's manual. The resulting DNA fragments were used as templates to PCR-amply the 5'-flanking sequences of the four P450 genes using the two general forward primers complementary to the adapter sequences and the two corresponding gene-specific reverse primers (see Additional file 1) for each P450 gene. The nested PCR reactions began with the primary PCR consisting of 25 cycles of 94°C denaturation for 2 min, 68°C annealing/extension for 4 min, followed by the secondary PCR consisting of 35 cycles of 94°C denaturation for 2 min and 68°C annealing/extension for 4 min. PCR products were run on a 1% agarose gel in 1×TAE buffer. The longest band for each gene was eluted from the gel using the QIAquick Gel Extraction Kit (Qiagen), and then directly cloned into the PGEM-T easy vector (Promega, MI). One white clone for each band was sequenced on Applied Biosystems 3730 DNA Analyzer twice in both directions using M13 forward and M13 reverse primers as well as internal primers designed on the basis of the determined sequences at the Genomic Analysis & Technology Core Facility of the University of Arizona.

### Cloning of *CYP9A12v3 *and *CYP9A14*

For cloning the *CYP9A12v3 *and *CYP9A14 *genomic sequences, two pairs of primers, CYP9A12F/CYP9A12R and CYP9A14F/CYP9A14R (see Additional file 1), were designed based on the cDNA sequences of their orthologs *CYP9A12 *[GenBank: AY371318] and *CYP9A14 *[GenBank: AY487948] in *H. armigera*, the Old World sibling species of *H. zea*. Their full length DNA sequences were then PCR-amplified with 35 cycles of 94°C denaturation for 2 min and 68°C annealing/extension for 4 min (*CYP9A12v3*) or 6min (*CYP9A14*). As described above, the PCR products obtained were cloned into the PGEM-T easy vector and sequenced.

### DNA sequence analysis

Sequences analyses were performed first by using blastn and blastx against a nonredundant database [69] and by using CENSOR against the Repbase [70] to scan the obtained *H. zea *P450 genomic sequences for TE insertions. The optimal global sequence alignment program was then used to further identify the sites of TE insertions, their TSDs, TIRs and other features by comparing a TE-free sequence and a TE-containing sequence [71]. DNA and protein sequence alignments were conducted using Genedoc software [72]. Sequences reported in this paper were deposited in the GenBank under the following accession numbers: Hztransib [GenBank: DQ788836], HzTc1 [GenBank: DQ788837], HzCR1 [GenBank: DQ788838], genomic DNA of *CYP9A12v3 *containing HzRTE-1-1 [GenBank: DQ788839], genomic DNA of *CYP9A14 *containing HzHzIS1-1, HzIS1-2, HzMITE2-1 and HzMITE3 [GenBank: DQ788840] and 5'-flanking sequence of *CYP321A1 *containing HzRTE-1-2, HzSINE1 and HzIS1-3 [GenBank: DQ788841].

### *In silico *mapping of TE insertions in the *Drosophila melanogaster *P450 genes

An extensive literature search was conducted to identify *D. melanogaster *P450s whose functions have been relatively defined. This led to characterization of eight insecticide resistance associated P450 genes (a DDT-resistant GST gene also included) and five ecdysone-synthesizing P450 genes (Table 2). A survey of TE insertions within or in close proximity (until encounter other genes or beyond 10 kb in both the 5' and 3' directions) to the thirteen P450 genes and the one GST gene was then conducted by *in silico *mapping at the flybase [26].

## Abbreviations

TE, transposable element; TIRs, terminal inverted repeats; TSDs, target site duplications; LINEs (long interspersed nuclear elements); SINEs (short interspersed nuclear elements); MITEs (miniature inverted-repeat transposable elements); LTR, long terminal repeat; EN, endonuclease; RT, reverse transcriptase; ORF, open reading frame; AP ENDO, apurinic-apyrimidic endonuclease

## Authors' contributions

SC and XL designed research. SC performed the research. SC and XL performed the analysis of the data set. XL and SC wrote the manuscript. All authors read and approved the final manuscript.

## Supplementary Material

Additional file 1 List of all primers used in the study. Click here for file

Additional file 2 Multiple sequences alignment of deduced truncated ORF of HzCR1 and related CR1 clade retrotransposons. The seven conserved RT subdomains are boxed and labeled 1 to 7. The names and accession numbers of the aligned sequences were: Maui [GenBank: AAD19348] from *Takifugu rubripes*, lines-fish [GenBank: BAE46429] and CR1-fish [GenBank: CAD32263] from *Danio rerio*, CR1 [GenBank: U88211] from *Gallus gallus*, Q [GenBank: U03849] from *Anopheles gambiae*, perere [GenBank: BK004067] from *Schistosoma mansoni*. Click here for file

Additional file 3 Multiple sequences alignment of deduced RT domain of HzRTE-1-1 and related RTE clade retrotransposons. The seven conserved RT subdomains are boxed and labeled 1 to 7. The names and accession numbers of the aligned sequences were: [GenBank: XM_783602] from *Strongylocentrotus purpuratus*, RTE-1 [GenBank: AF054983] and RTE-2 [GenBank: U00063] from *Caenorhabditis elegans*, SR2 [GenBank: AF025672] and SR3 [GenBank: DQ008121] from *Schistosoma mansoni*, Bov-B LINE [GenBank: AF332697] from *Vipera ammodytes*. Click here for file
