# Supplementary material for: Transposable elements are enriched within or in close proximity to xenobiotic-metabolizing cytochrome P450 genes
Source: BMC Evol Biol. 2007 Mar 23;7:46. doi: 10.1186/1471-2148-7-46 (PMC1852546; doi:10.1186/1471-2148-7-46)
Supplement: Additional file 1 — List of all primers used in the study. [file 1471-2148-7-46-S1.doc]

List of all primers used in this study

| **primer name** | **primer sequence (5'-3')** |
| --- | --- |
| 9A12F | ATGATACTAGTCCTGGTCTGGGTG |
| 9A12R | CTACTGCCTAGGTCTGAATCTAAG |
| 9A14F | ATGATAGCCCTACTATGGCTGGCG |
| 9A14R | TTACTGGCGCAGCTTGACCCTAAT |
| Hz6B8GSP1 | ATAAATAAAGGGTAACGATTAACACTG |
| Hz6B8GSP2 | ATCTCGAGGATAGCACTGCCGGTAGATATAAGACC |
| Hz6B27GSP1 | CCTTGTGAAATAAAAATAAAGAGTGACG |
| Hz6B27GSP2 | ATCTCGAGGATATCACTGCCGGAAAATAAAAGATCC |
| Hz321A1GSP1 | ACCGATCAGGTACCACGTTAGTAAGAG |
| Hz321A1GSP2 | ATCTCGAGCCTAATAAAATCAGTGGTAGTTGTAAC |
| Hz321A2GSP1 | TGATTCGGACTCAGAATGTGTCAAAGC |
| Hz321A2GSP2 | ATCTCGAGCTATCAGAACATGGCTACAGACCTACT |
