# Supplementary figures and images for: Transposable elements are enriched within or in close proximity to xenobiotic-metabolizing cytochrome P450 genes
Source: BMC Evol Biol. 2007 Mar 23;7:46. doi: 10.1186/1471-2148-7-46 (PMC1852546; doi:10.1186/1471-2148-7-46)

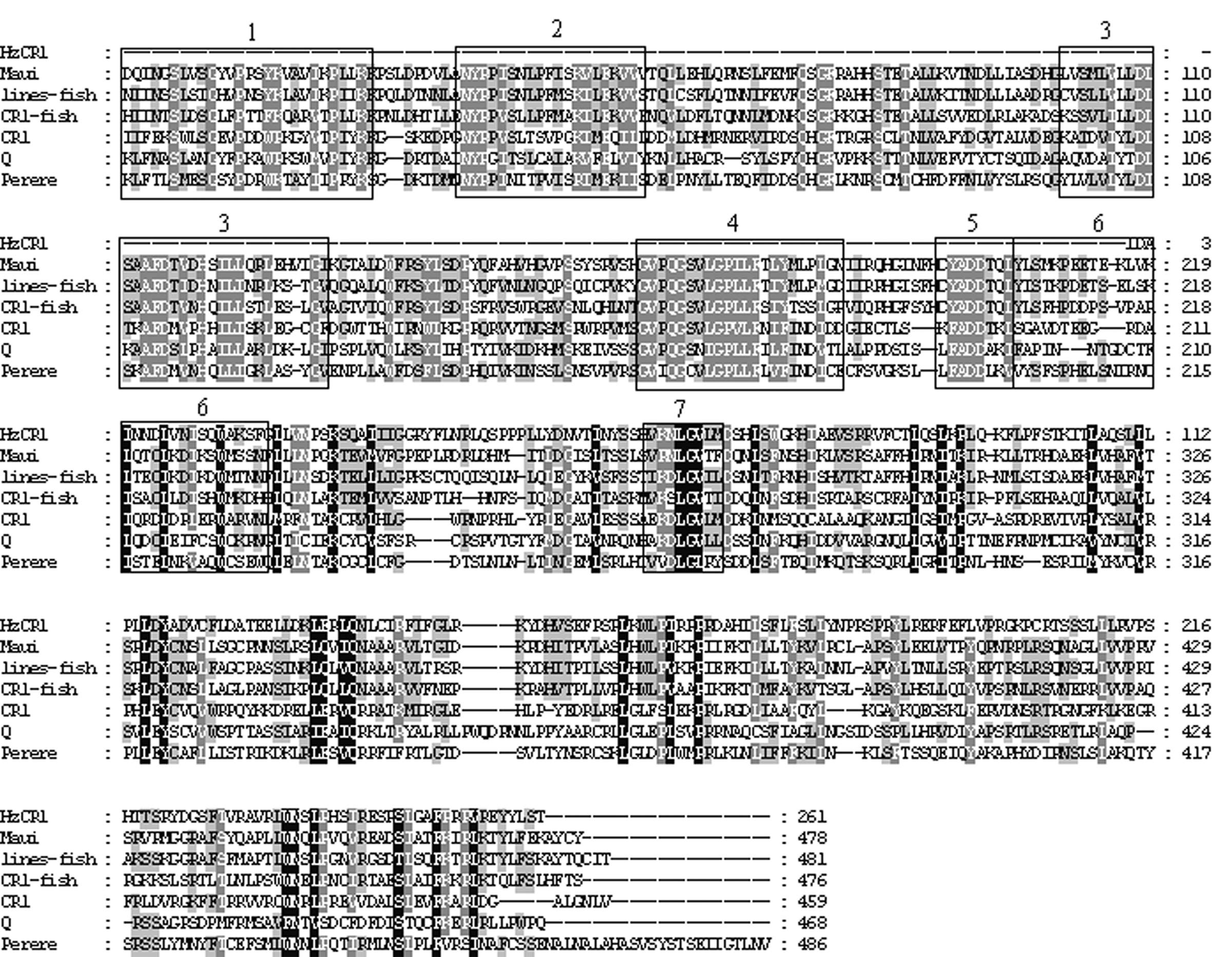

Supplement: Additional file 2 — Multiple sequences alignment of deduced truncated ORF of HzCR1 and related CR1 clade retrotransposons. The seven conserved RT subdomains are boxed and labeled 1 to 7. The names and accession numbers of the aligned sequences were: Maui [GenBank: AAD19348] from Takifugu rubripes, lines-fish [GenBank: BAE46429] and CR1-fish [GenBank: CAD32263] from Danio rerio, CR1 [GenBank: U88211] from Gallus gallus, Q [GenBank: U03849] from Anopheles gambiae, perere [GenBank: BK004067] from Schistosoma mansoni. [file 1471-2148-7-46-S2.tiff]

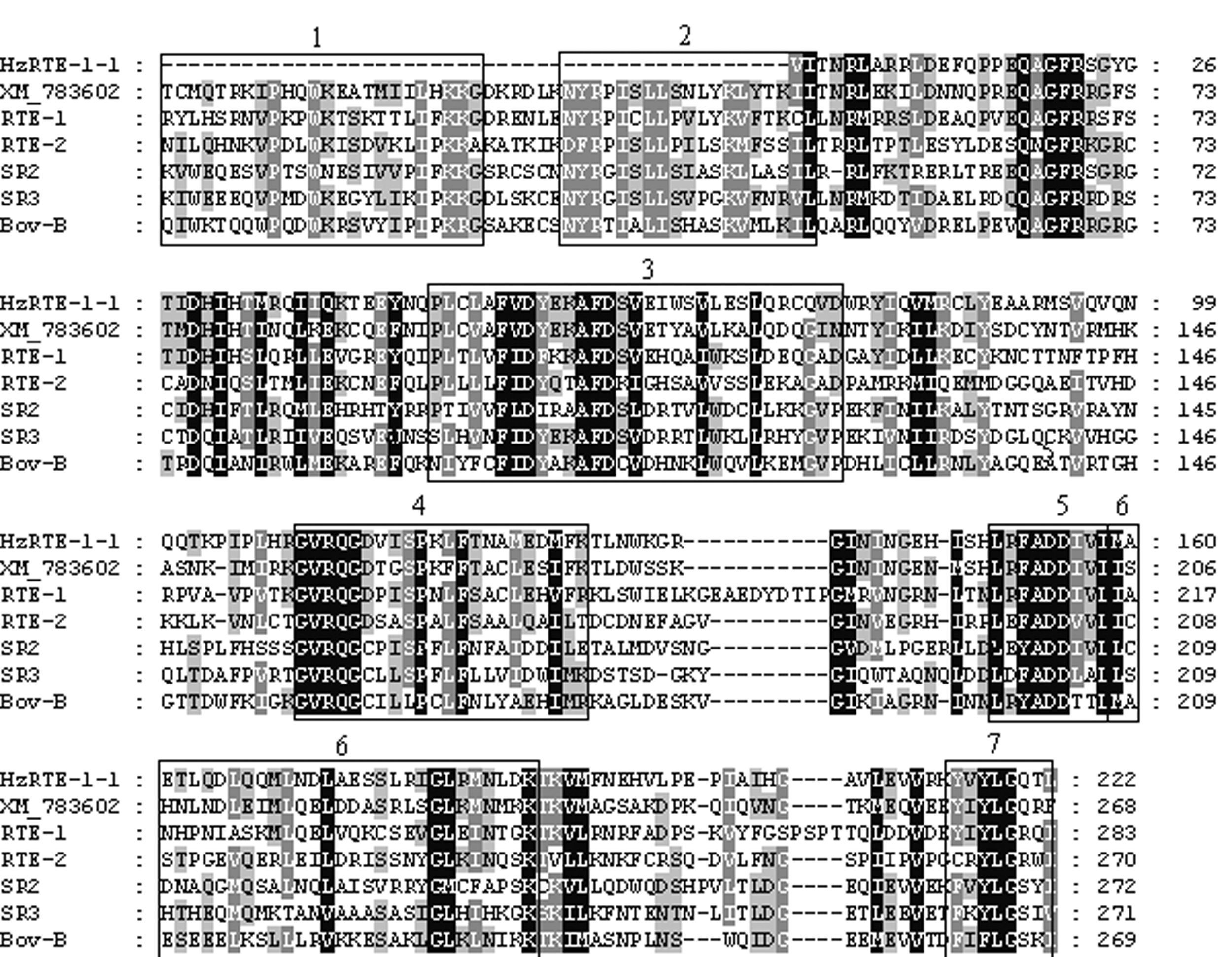

Supplement: Additional file 3 — Multiple sequences alignment of deduced RT domain of HzRTE-1-1 and related RTE clade retrotransposons. The seven conserved RT subdomains are boxed and labeled 1 to 7. The names and accession numbers of the aligned sequences were: [GenBank: XM_783602] from Strongylocentrotus purpuratus, RTE-1 [GenBank: AF054983] and RTE-2 [GenBank: U00063] from Caenorhabditis elegans, SR2 [GenBank: AF025672] and SR3 [GenBank: DQ008121] from Schistosoma mansoni, Bov-B LINE [GenBank: AF332697] from Vipera ammodytes. [file 1471-2148-7-46-S3.tiff]
